# Efficacy of Endoscopic Ultrasound‐guided Fine Needle Biopsy With Simplified Macroscopic On‐site Evaluation (With Video)

**DOI:** 10.1002/deo2.70347

**Published:** 2026-06-27

**Authors:** Kento Shionoya, Ryosuke Tonozuka, Shuntaro Mukai, Yuki Joyama, Takayoshi Tsuchiya, Reina Tanaka, Kenjiro Yamamoto, Kazumasa Nagai, Yukitoshi Matsunami, Hiroyuki Kojima, Hirohito Minami, Noriyuki Hirakawa, Kyoko Asano, Takao Itoi

**Affiliations:** ^1^ Department of Gastroenterology and Hepatology Tokyo Medical University Tokyo Japan

**Keywords:** endoscopic ultrasound‐guided fine needle biopsy, endoscopic ultrasound‐guided tissue acquisition, macroscopic on‐site evaluation, pancreatic cancer, S‐MOSE

## Abstract

**Background and Aims:**

With advances in preoperative chemotherapy and comprehensive genomic profiling, endoscopic ultrasound‐guided fine needle biopsy (EUS‐FNB) has become vital for diagnosing pancreatic and peridigestive tract lesions. However, evidence remains limited regarding the performance of new‐generation puncture needles and simplified specimen evaluation methods. This study retrospectively assessed the diagnostic ability and procedural adverse events (AEs) of a new‐generation puncture needle for solid pancreatic, subepithelial, and other lesions.

**Methods:**

We analyzed data from 1232 consecutive patients who underwent EUS‐FNB between August 2016 and November 2023 at a single care center. Specimen processing was performed using the simplified macroscopic on‐site evaluation (S‐MOSE) method, which does not require additional pathological tests such as rapid on‐site evaluation or cytology.

**Results:**

The overall diagnostic accuracy using S‐MOSE was 93.7%. Specifically, the diagnostic accuracy for pancreatic cancer was 95.8%. The AE rate was 1.1% (14/1232), including pancreatitis (six), hemorrhage (five), hematoma (two), and pancreatic fistulas (one). The first puncture diagnostic rate was 83.6%, which increased to 92.5% after the second puncture, with only a marginal gain of 1.2% beyond the third puncture. Multivariable analysis identified smaller lesion size and benign pathology as factors associated with lower diagnostic accuracy; conversely, malignant pathology was associated with higher diagnostic accuracy (odds ratio 1.895, *p* = 0.011).

**Conclusion:**

EUS‐FNB with S‐MOSE, typically requiring up to two punctures, provides efficient and accurate histopathological diagnosis across diverse lesions while minimizing procedural burden.

**Trial Registration**: N/A.

## 1 Introduction

Endoscopic ultrasound‐guided tissue acquisition (EUS‐TA) is widely used for diagnosing pancreatic lesions [1]. In pancreatic cancer (PC), the increasing use of preoperative neoadjuvant chemotherapy and comprehensive genomic profiling has made definitive tissue diagnosis and the collection of adequate tumor‐containing samples essential [2, 3]. Various efforts aim to increase tissue collection volume and reduce the number of punctures. EUS‐guided fine‐needle biopsy (EUS‐FNB) offers better accuracy than EUS‐guided fine‐needle aspiration (EUS‐FNA), facilitating an increased tissue volume while decreasing the number of punctures [4, 5, 6, 7, 8]. Recently, new‐generation EUS‐FNB needles have been developed to increase tissue yield while preserving tissue structure. Various EUS‐FNB needle tip designs, such as the “Franseen” or “Fork‐tip”, have been introduced, improving the accuracy compared to conventional lancet‐type needles [9, 10, 11, 12]. However, the puncture performance and tissue collection capabilities of these needles have not been thoroughly investigated. Furthermore, approaches such as rapid on‐site evaluation (ROSE) and macroscopic on‐site evaluation (MOSE) for subepithelial lesions (SELs) further enhance accuracy and specimen quality [13, 14]. However, not all facilities can perform ROSE because of limited cytology support, and MOSE specimen processing is relatively complex. Furthermore, with the advancement of FNB needles, the significance of additional procedures has diminished. EUS‐FNB is also valuable for diagnosing peridigestive tract lesions, such as the biliary tract, liver, and lymph node (LN). Specific neoplasms, including malignant lymphoma and intra‐abdominal tumors, can be challenging to diagnose based solely on imaging findings. Therefore, immunohistochemical staining, morphological examination, and genetic testing are often performed, underscoring the necessity of histological sampling [15, 16].

To further enhance procedural efficiency, we developed a novel technique to facilitate tissue acquisition assessment. This study aimed to retrospectively examine the diagnostic performance and procedural adverse events (AEs) of EUS‐FNB using simplified MOSE (S‐MOSE). The analysis included diverse lesions, thereby validating S‐MOSE as a reliable and simplified approach for assessing tissue adequacy in a large cohort.

## 2 Methods

### 2.1 Patients

This retrospective cohort study analyzed data from 1232 consecutive patients who underwent EUS‐FNB using our simplified tissue processing method between August 2016 and November 2023 at a single care center. Inclusion criteria were: (1) age ≥18 years; and (2) EUS‐FNB performed with one of four types of needles for solid lesions. Exclusion criteria were: (1) age < 18 years; and (2) patients who opted out of the study.

We used four needle types: three symmetric heel Franseen needles (Acquire; Boston Scientific Corp., MA, USA), a crown cut Franseen needle (SonoTip TopGain; Medi‐Globe GmbH., Bayern, Germany), a fork‐tip needle (Sharkcore; Medtronic plc, Dublin, Ireland), and a three‐prong asymmetric tip needle (Trident; MicroTech Endoscopy; Nanjing, China).

### 2.2 Novel SMOSE for EUS‐FNB

**FIGURE 1:**
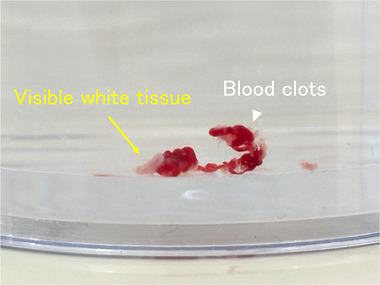
Sufficient tissue collection with simplified‐macroscopic on‐site evaluation (S‐MOSE). A white specimen ≥ 0.5 mm in length was obtained in the formalin bottle and judged sufficient for tissue collection using S‐MOSE. (↑) Sufficient visible white tissue collection was observed. (△) Blood clots.

The target lesion was visualized from the gastrointestinal (GI) tract using a curved linear‐array EUS endoscope (GF‐UCT‐260; Olympus, Tokyo, Japan). After confirming the absence of vascular or other organ involvement, the lesion was punctured using a 19/22/25‐gauge needle. For patients on antiplatelet or antithrombotic medications, EUS‐FNB was performed after appropriate drug withdrawal or replacement based on Japanese guidelines [17]. The stylet was removed, and 20 mL of negative syringe suction was applied during the first puncture. If extensive macroscopic blood contamination was observed, either the slow‐pull technique or no suction was applied during the second puncture [18]. The needle was advanced and retracted within the lesion approximately 10 times. The obtained tissue specimens were immediately fixed in 10% neutral‐buffered formalin for histological examination. The number of passes was determined based on the presence of macroscopically visible core specimens, defined as white or yellow tissue fragments clearly visible without employing ROSE, MOSE, or cytology. This procedure is called S‐MOSE. After removing the needle, its lumen was extruded using a 20 mL air‐filled syringe. After expelling 20 mL, the stylet was reinserted into the inner tube, and the remaining tissue was pushed into the formalin container. The lid of the container was placed onto the syringe to confirm the presence of white tissue. Passes were repeated until adequate macroscopically visible core tissue was confirmed by the endoscopist and nurse (Figure 1 and Video S1).

### 2.3 Tissue Specimen Handling

The fixed tissue specimens were routinely processed and embedded in paraffin in a histological tissue specimen handling room. The paraffin‐embedded tissues were cut into 3‐µm slices, and only sections predominantly containing tissue specimens were processed into slides. Tissue sections were stained with hematoxylin and eosin. Immunohistochemical staining was added as needed.

### 2.4 Outcomes and Definitions

The study outcomes included (1) specimen adequacy, (2) the accuracy of the final pathological diagnosis, and (3) AEs. Specimen adequacy was defined as the successful acquisition of tissue sufficient for histological evaluation. Pathological diagnosis from surgical specimens was considered the final diagnosis for patients who underwent surgery for suspected malignant diseases. Additional staining was also performed on resected specimens as needed. Diagnostic accuracy was primarily assessed in surgically confirmed cases, in which the EUS‐FNB diagnosis was compared with the surgical pathology. In non‐surgical cases, because a definitive disease‐specific reference standard was not always available, the clinical usefulness of EUS‐FNB was interpreted with caution based on follow‐up imaging findings and clinical course for >six months. Therefore, in such cases, the results were interpreted mainly in terms of tissue acquisition and clinically relevant diagnostic categorization rather than strict discrimination among all individual disease entities. Cases without a definitive diagnosis were followed with repeat EUS‐FNB or imaging, as clinically indicated. AEs were defined as any procedure‐related events [19, 20]. Bleeding was defined as a clinically evident hemorrhage, such as melena or endoscopically confirmed bleeding requiring clinical observation or intervention, whereas hematoma was defined as a localized blood collection detected radiologically without overt GI bleeding. Post‐procedural pancreatitis was defined according to the revised Atlanta classification [21]. Pancreatic fistula was defined as radiologic evidence of pancreatic fistula or a new peripancreatic fluid collection not meeting the criteria for acute pancreatitis. Sedation‐related AEs and other procedure‐associated events, such as aspiration pneumonia, were not systematically assessed in this study and were therefore not included in the analysis. Trainees were defined as physicians with <5 years of EUS‐TA procedure experience, and experts were defined with >5 years. Patients at risk of bleeding were defined as those with cirrhosis, dialysis, or hematologic disorders associated with bleeding risk.

### 2.5 Statistical Analysis

Continuous variables were presented as medians and interquartile ranges (IQRs), and categorical variables were presented as counts with percentages. To identify factors associated with the diagnostic yield of EUS‐FNB, we first performed univariate logistic regression. Variables with *p* < 0.25 were retained for multivariate modeling using a purposeful selection procedure [22, 23], whereby covariates were retained in the model if they remained significant (*p* ≤ 0.05) or their removal changed the odds ratio (OR) of key covariates by ≥20%. The final model is presented with ORs and 95% confidence intervals (CIs). In addition, subgroup diagnostic accuracy was calculated as the proportion of lesions in each category for which the biopsy result matched the final diagnosis. To assess lesion size, multiple cutoffs (15, 20, 25, and 35 mm) were explored using separate logistic regression models, and the best cutoff was selected based on the Akaike Information Criterion (AIC).

AEs were analyzed using Fisher's exact tests for baseline (age and sex), clinical (malignancy, lesion size, and location), and procedural (route, needle, gauge, and operator experience) factors, with p‐values adjusted for multiple comparisons using the Benjamini–Hochberg (BH) procedure [24]. Relative risks (RRs) and 95% CIs were also calculated. A two‐sided *p* ≤ 0.05 was considered statistically significant. All analyses were performed using the R software (version 4.4.2; The R Foundation for Statistical Computing, Vienna, Austria).

## 3 Results

### 3.1 Patient Characteristics

Table 1 summarizes the characteristics of the study population. 1232 patients were included with a median age of 68.5 years (IQR 58–75). Note that 735 (60%) were male and 497 (40%) were female. Most target lesions were located in the pancreas (944, 77%), followed by SELs (123, 10%), LNs (93, 7.5%), and others (72, 5.8%). Final diagnoses were benign in 276 (22%) and malignant in 956 (78%) (Table 2). The median lesion size was 23.3 mm (IQR 17–30). 22‐gauge needles were most frequently used (1129 cases, 92%), with one or two passes in most cases (369 and 736 patients, respectively). Franseen needles were used in 1000 cases (81%), and the most common puncture routes were the stomach (665, 54%) and duodenum (529, 43%).

**TABLE 1:** Patient characteristics.

Characteristic	*N* = 1232
Age (years)	68.5 [58–75]
Sex	
Female	497 (40)
Male	735 (60)
Location of the lesion	
Pancreatic	944 (77)
Subepithelial	123 (10)
Lymph node	93 (7.5)
Others	72 (5.8)
Malignancy	
Benign	276 (22)
Malignant	956 (78)
Lesion size (mm)	23.3 [17–30]
Needle type	
Acquire	1000 (81)
Sharkcore	54 (4.4)
Sonotip Top‐gain	60 (4.9)
Trident	118 (9.6)
Puncture route	
Duodenum	529 (43)
Esophagus	28 (2.3)
Jejunum	4 (0.3)
Rectum	6 (0.5)
Stomach	665 (54)
Needle gauge	
19	10 (0.8)
22	1,129 (92)
25	93 (7.5)
Number of passes	
1	369 (30)
2	736 (60)
3	118 (9.6)
4	8 (0.6)
5	0
6	1 (<0.1)
Median [IQR]; *n* (%)	

Abbreviation: IQR, Interquartile range.

**TABLE 2:** Final diagnoses from endoscopic tissue acquisition using simplified macroscopic on‐site evaluation.

	*N* = 1232					
	Malignant (*N* = 956)			Benign (*N* = 276)		
Final diagnosis	**Pancreas**	Pancreatic cancer	664	**Pancreas**	Autoimmune pancreatitis	77
		Pancreatic ductal cancer	645		Chronic pancreatitis	60
		Adenosquamous carcinoma	11		Benign pancreatic tissue	22
		Anaplastic carcinoma	5		Serous cyst neoplasm	6
		Acinar cell carcinoma	2		Accessory spleen	6
		Mucinous adenocarcinoma	1		Schwannoma	2
		Pancreatic neuroendocrine neoplasm	61		Solitary fibrous tumor	2
		Solid pseudopapillary neoplasm	20		Fat deposition	1
		Metastases of other organ cancers	10		Fibrosis	1
		Neuroendocrine carcinoma	4	**Subepithelial lesion**	Leiomyoma	27
		Intraductal Papillary Mucinous Carcinoma	3		Benign	13
		Gastrointestinal stromal tumor	2		Schwannoma	8
		Bile duct cancer	1		Ectopic pancreas	7
		Anaplastic or pleomorphic sarcoma	1		Lipoma	1
		Paraganglioma	1		Spindle cell tumor	1
	**Subepithelial lesion**	Gastrointestinal stromal tumor	56		Focal nodular hyperplasia	1
		Sarcoma	3		Hamartoma	1
		Neuroendocrine tumor	2	**Others**	Benign lymphoid tissue (lymph node)	19
		Pancreatic ductal cancer	1		Retroperitoneal fibrosis	3
		Gastric cancer	1		Xanthogranulomatous cholecystitis	3
		Malignant lymphoma	1		Primary Sclerosing Cholangitis	2
	**Others**	Metastases of cancer	38		Schwannoma	2
		Malignant lymphoma	24		Spindle cell tumor	1
		Gallbladder cancer	17		Fibrosis	1
		Gastric cancer	12		Ectopic pancreas	1
		Gastrointestinal stromal tumor	10		Sarcoidosis	1
		Sarcoma	3		Accessory spleen	1
		Adenocarcinoma of unknown primary	3		Liver abscess	1
		Leiomyosarcoma	3		Inflammatory pseudotumor (Liver)	1
		Bile duct cancer	3		Hyperplasia of the Brunner gland	1
		Duodenum cancer	2		Subacute necrotizing lymphadenitis	1
		Cholangiocellular carcinoma	3		Reactive lymphadenopathy	1
		Rectal cancer	2		Lipoma	1
		Neuroendocrine carcinoma (Rectum)	1			
		Neuroendocrine carcinoma (gallbladder)	1			
		Liposarcoma	1			
		Malignant mesothelioma	1			
		Hepatocellular carcinoma	1			

### 3.2 Adequate Tissue Acquisition

The rate of adequate tissue acquisition was 97.6% (1202/1232).

### 3.3 Overall Diagnostic Accuracy

**FIGURE 2:**
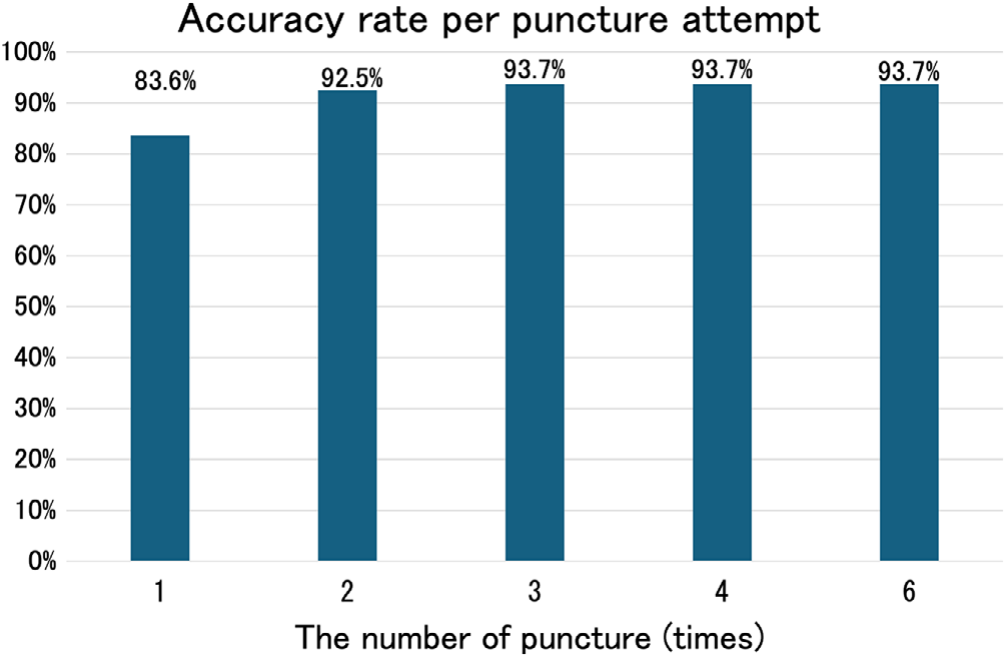
Additive effect of the number of punctures on diagnostic accuracy. In all cases, 83.6% of patients were correctly diagnosed after one puncture and 92.5% after the second puncture. The additional diagnostic effect of the third and subsequent punctures was approximately 1.2%.

The overall accuracy was 93.7% (1154/1232). In 89.7% (1105/1232), the procedure was completed within two punctures, achieving an accuracy of 93.6%. The accuracy for PC was 95.8%. Across all cases, 83.6% of patients were correctly diagnosed after one puncture and 92.5% after two punctures (Table 3). The additional effect of the third and subsequent punctures was approximately 1.2%, with minimal additional effect even after ≥4 punctures (Figure 2).

**TABLE 3:** Factors associated with diagnostic accuracy by univariable and multivariable logistic regression.

	Accuracy	Crude OR (95% CI)	*p*‐Value	Adjusted OR (95% CI)^*^	*p*‐Value
Overall	93.7% (1154/1232)				
Age (years)		1.013 (0.997–1.031)	0.108		
Sex					
Female	94.2% (468/497)	1 (reference)			
Male	93.3% (686/735)	0.868 (0.534–1.384)	0.557		
Malignancy					
Benign	88.4% (244/276)	1 (reference)		1 (reference)	
Malignant	95.2% (910/956)	2.581 (1.597–4.126)	<0.001	1.895 (1.146–3.096)	0.011
Pancreatic cancer	95.8% (636/664)				
Lesion size (mm)		1.056 (1.030–1.085)	<0.001	1.036 (1.010–1.064)	0.008
Location of the lesion					
Pancreatic	94.3% (890/944)	1 (reference)			
Subepithelial	90.2% (111/123)	0.561 (0.301–1.129)	0.084		
Lymph node	92.5% (86/93)	0.745 (0.350–1.843)	0.481		
Others	93.1% (67/72)	0.813 (0.344–2.392)	0.669		
Puncture route					
Duodenum/jejunum/rectum	92.4% (498/539)	1 (reference)			
Esophagus/stomach	94.7% (656/693)	1.460 (0.922–2.319)	0.107		
Needle type					
Franseen (Acquire/SonoTip TopGain)	94.2% (999/1060)	1 (reference)			
Others (Trident/SharkCore)	90.1% (155/172)	2.661 (0.972–10.981)	0.101		
Needle gauge					
19/22	94.9% (1080/1139)	1 (reference)		1 (reference)	
25	79.6% (74/93)	0.213 (0.122–0.383)	<0.001	0.327 (0.181–0.612)	<0.001
Number of passes					
1	90.8% (335/369)	1 (reference)			
2	95.0% (699/736)	1.917 (1.179–3.111)	0.008		
≥3	94.5% (120/127)	1.740 (0.795–4.373)	0.196		
Operators					
Trainees	94.5% (651/689)	1 (reference)			
Experts	92.6% (503/543)	0.734 (0.463–1.163)	0.187		

Abbreviation: OR, odds ratio.

* Adjusted for malignancy, size, and needle gauge.

In univariate analysis, malignant lesions, larger lesion size, and smaller needle gauges (larger diameter) were significantly associated with higher accuracy. After multivariate adjustment for malignancy, lesion size, and needle gauge, both malignancy (adjusted OR [aOR] 1.895, 95% CI, 1.146–3.096; *p* = 0.011) and larger lesion size (aOR 1.036, 95% CI, 1.010–1.064; *p* = 0.008) remained significant for accurate diagnosis. No evidence of collinearity or interaction between malignancy and lesion size was observed in the final model. Needle gauge also significantly influenced diagnostic performance. Using 25‐gauge needles was associated with lower accuracy than using 19/22‐gauge needles (aOR 0.327, 95% CI, 0.181–0.612; *p* < 0.001). In contrast, other factors‐such as lesion location, puncture route, needle type, number of passes, and operator were not included in the final model based on predefined criteria (data not shown).

### 3.4 Diagnostic Accuracy by Lesion Size

Table 4 summarizes the odds of achieving an accurate diagnosis across stratified lesion size categories. Based on the AIC, the optimal cutoff was 20 mm, followed by 15 mm. Accuracy increased significantly with larger lesion diameters, rising from 87.0% for lesions <15 mm to 97.2% for lesions >35 mm. Compared to lesions <15 mm, the odds of an accurate diagnosis were significantly higher for lesions ≥25 mm (aOR 2.197, 95% CI 1.086–4.540; *p* = 0.030 for 25–<35 mm; aOR 3.187, 95% CI 1.341–8.451; *p* = 0.012 for >35 mm).

**TABLE 4:** Diagnostic accuracy and odds ratios for lesion size categories and cutoff values.

Lesion size (mm)	Accuracy	Crude OR (95% CI)	*p*‐value	Adjusted OR (95% CI)^*^	*p*‐Value
<15	87.0% (160/184)	1 (reference)		1 (reference)	
15 to <20	90.5% (191/211)	1.433 (0.764–2.711)	0.263	1.219 (0.636–2.349)	0.550
20 to <25	95.1% (232/244)	2.900 (1.436–6.159)	0.004	2.030 (0.970–4.427)	0.065
25 to <35	95.6% (328/343)	3.280 (1.691–6.555)	<0.001	2.197 (1.086–4.540)	0.030
>35	97.2% (243/250)	5.207 (2.303–13.346)	<0.001	3.187 (1.341–8.451)	0.012
**Cutoffs**					
20 mm					
<20 mm	88.9% (351/395)	1 (reference)		1 (reference)	
≥20 mm	96.0% (803/837)	2.961 (1.865–4.740)	<0.001	2.100 (1.272–3.473)	0.004
15 mm					
<15 mm	87.0% (160/184)	1 (reference)		1 (reference)	
≥15 mm	94.8% (994/1048)	2.761 (1.635–4.544)	<0.001	1.846 (1.047–3.159)	0.029

Abbreviations: CI, Confidence interval; OR, Odds ratio.

* Adjusted for malignancy, size, and needle gauge.

Using 20 mm as the threshold, lesions ≥ 20 mm had significantly higher accuracy than those <20 mm (96.0% vs. 88.9%; aOR 2.100, 95% CI 1.272–3.473; *p* = 0.004). Similarly, applying a 15 mm threshold, lesions ≥15 mm also demonstrated higher accuracy (94.8% vs. 87.0%; aOR 1.846, 95% CI 1.047–3.159; *p* = 0.029).

### 3.5 Adverse Events

The overall AE rate was 1.1% (14/1232), including pancreatitis (six), hemorrhage (five), hematoma (two), and pancreatic fistulas (one). The patient with pancreatic fistula had a tumor caused by autoimmune pancreatitis (AIP). One bleeding case required clip hemostasis; however, the patient improved conservatively, and no deaths occurred. No significant increase in AEs was observed among older patients (≥ 65 years, RR 1.6, 95% CI 0.5–5.2) or those with malignant lesions (RR 0.5, 95% CI 0.2–1.5). Needle type, gauge (Franseen vs. others; 25‐gauge vs. 19/22‐gauge), number of passes, and operator were also not associated with a higher complication rate after adjusting for multiple comparisons (all BH‐adjusted *p* > 0.05). No AEs occurred among patients who underwent ≥3 passes, had lesions categorized as “other” (not pancreatic, subepithelial, or LN), or received 25‐gauge needles; however, these results should be interpreted with caution, given the relatively small sample sizes in these subgroups (Table 5).

**TABLE 5:** Procedure‐related adverse events.

	Risk of adverse event	RR (95% CI)	*p*‐Value
Overall	1.1% (14/1232)		
Age (years)			0.584
<65	0.8% (4/487)	1 (reference)	
≥65	1.3% (10/745)	1.6 (0.5–5.2)	
Sex			0.791
Female	1.0% (5/497)	1 (reference)	
Male	1.2% (9/735)	1.2 (0.4–3.6)	
Malignancy			0.329
Benign	1.8% (5/276)	1 (reference)	
Malignant	0.9% (9/956)	0.5 (0.2–1.5)	
Lesion size (mm)			0.567
<20	0.8% (3/395)	1 (reference)	
≥20	1.3% (11/837)	1.7 (0.5–6.2)	
Location of the lesion			>0.99
Pancreatic	1.3% (12/944)	1 (reference)	
Subepithelial	0.8% (1/123)	0.6 (0.1–4.9)	
Lymph node	1.1% (1/93)	0.8 (0.1–6.4)	
Others	0% (0/72)		
Puncture route			0.290
Duodenum/jejunum/rectum	0.7% (4/539)	1 (reference)	
Esophagus/stomach	1.4% (10/693)	1.9 (0.6–6.2)	
Needle type			0.706
Franseen (Acquire/SonoTip TopGain)	1.2% (13/1060)	1 (reference)	
Others (Trident/SharkCore)	0.6% (1/172)	0.5 (0.06‐3.6)	
Needle gauge			0.617
19/22	1.2% (14/1139)		
25	0% (0/93)		
Number of passes			0.516
1	1.1% (4/369)	1 (reference)	
2	1.4% (10/736)	1.3 (0.4–4.0)	
≥3	0% (0/127)		
Operators			0.419
Trainees	0.9% (6/689)	1 (reference)	
Experts	1.5% (8/543)	1.7 (0.6–4.8)	

Abbreviation: CI, Confidence interval; RR, Risk ratio.

* Fisher's exact test

## 4 Discussion

Recent advancements in chemotherapy or operation have increased the need for tissue confirmation and adequate sampling in PC [25, 26]. EUS‐TA is also important for SELs or other lesions, where immunohistochemistry and molecular testing may guide management [15, 16]. In this large cohort (>1200 cases), EUS‐FNB with S‐MOSE achieved high diagnostic performance with a low AE rate. Reported diagnostic accuracy of EUS‐FNB for PC is high (86%–97%) [7, 27, 28, 29, 30], and recent RCTs suggest that EUS‐FNB alone can provide sufficient core tissue while reducing procedural complexity [31, 32, 33, 34, 35]. We observed comparable performance across needle designs [36]. Diagnostic accuracy increased with lesion size, with lower performance in lesions ≤20 mm, likely reflecting technical difficulty and limited tissue acquisition in small targets. The lesion size cutoff was selected by comparing several candidate thresholds using the AIC and should therefore be regarded as exploratory. Accordingly, the possibility of overfitting cannot be excluded.

Reports on the number of punctures required for accurate diagnosis have varied, with estimates suggesting that 70%–90% of cases require two punctures [37, 38]. The European Society of Gastrointestinal Endoscopy recommends two to three passes to ensure at least 90% accuracy for malignancy [13, 39, 40, 41]. In this study, two punctures were adequate across all lesions, regardless of needle type. Although a single pass yielded an accuracy of 83.6%, this increased to 92.5% after the second puncture. The additional diagnostic impact beyond two passes was approximately 1.2%. An accuracy of 93.7% indicated that S‐MOSE with two punctures sufficiently confirmed tissue adequacy for diagnosis. Because the primary outcome was diagnostic accuracy, this measure may partly reflect tissue acquisition and histological interpretability. In non‐surgical cases, the results should be interpreted cautiously because a definitive disease‐specific reference standard was not always available. The number of passes was determined based on the presence of macroscopically visible specimens. However, when the specimen contains substantial blood contamination, accurate diagnosis may still be difficult despite an apparently sufficient specimen volume.

EUS‐FNB is increasingly being performed for lesions outside the pancreas. Although most SELs are benign, some tumors may become malignant, highlighting the importance of acquiring appropriate specimens for diagnosis. Furthermore, as diagnosis often requires immunohistochemical analysis, sufficient tissue acquisition is essential. Nonetheless, even with the use of jumbo biopsy forceps and the “bite‐on‐bite” biopsy technique, the diagnostic yield of endoscopic biopsy tends to be low (17%–59%), with bleeding complications reported in 35% [42]. Conversely, EUS‐TA has shown higher accuracy for SELs (60%–80%) [43]. EUS‐TA is also performed on LNs, biliary tree, and retroperitoneal lesions when histological diagnosis may affect therapeutic management or when other examinations fail to provide adequate information. Notably, EUS‐TA is reportedly safer than conventional percutaneous methods for diagnosing or evaluating liver diseases [44]. The usefulness of LN or peritoneal lesions assessment has been demonstrated [45, 46]. Despite the need for immunostaining for diagnosis and the large amount of tissue required, puncture is challenging, and the accuracy rate is considered low [47]. In this study, accuracy for outside pancreas lesions was over than 90%, demonstrating that EUS‐FNB with S‐MOSE is effective for diagnosing. Overall, factors contributing to nondiagnostic outcomes included benign pathology and smaller lesion size. Previous studies have shown that lesion location is a factor influencing diagnostic difficulty; however, this association was not observed.

AEs associated with EUS‐TA include bleeding and pancreatitis, with reported rates ranging from 0.59% to 2.1% [19, 37, 44, 48, 49]. In this study, 14 patients (1.1%) experienced AEs. Procedural factors (needle type, passes, operator) were not associated with a higher AE rate after adjustment for multiple comparisons (all BH‐adjusted *p* > 0.05). No AEs were observed among patients who underwent ≥ three passes, had lesions categorized as “other” (not pancreatic, subepithelial, or LN), or received 25‐gauge needles; however, these results should be interpreted cautiously given the relatively small sample sizes in these subgroups. One AIP patient had a moderate pancreatic fistula, representing a rate of 0.08%. Patients with AIP may have a higher incidence of pancreatitis and pancreatic fistulas than those without non‐AIP, likely because AIP retains normal pancreatic tissue, unlike PC. Previous reports have highlighted a higher incidence of AEs when small lesions involve the normal pancreatic parenchyma during puncture [50, 51]. Regarding bleeding, risk was unaffected by the presence of bleeding predispositions or use of antiplatelet/antithrombotic medications. Although careful monitoring is crucial, procedures can be performed safely when these medications are appropriately managed following the Japanese guidelines [17]. No significant differences were observed in AE rates based on needle size. Although AIP was identified as an independent risk factor for, punctures can be performed safely with sufficient monitoring, even when bleeding risk factors exist, or treatments involve antithrombotic drugs.

To the best of our knowledge, this is the first study to examine the efficacy of EUS‐FNB with S‐MOSE for any lesions. However, this study had some limitations. First, it was a retrospective study conducted at a single facility. Second, the occurrence of SELs and other lesions was limited. Finally, relatively few cases using 25/19‐gauge needles were included. Future RCTs with larger sample sizes, direct comparisons of new‐generation EUS‐FNB needles, various needle sizes, and alternative puncture techniques are warranted.

In conclusion, EUS‐FNB with S‐MOSE typically requires up to two punctures, provides an efficient and accurate histopathological diagnosis across diverse lesions, while minimizing procedural burden.

## Author Contributions

**Kento Shionoya**, **Ryosuke Tonozuka**, and **Shuntaro Mukai**: prepared the manuscript. **Kento Shionoya** and **Yuki Joyama**: performed the statistical analysis. **Kento Shionoya**, **Ryosuke Tonozuka**, **Shuntaro Mukai**, **Takayoshi Tsuchiya**, **Reina Tanaka**, **Kenjiro Yamamoto**, **Kazumasa Nagai**, **Yukitoshi Matsunami**, **Hiroyuki Kojima**, **Hirohito Minami**, **Noriyuki Hirakawa**, and **Kyoko Asano**: managed the patients. **Takao Itoi**: conducted the correspondence. All the authors have read and approved the final version of the manuscript.

## Funding

The authors have nothing to report.

## Conflicts of Interest

The authors declare no conflicts of interest.

## Ethics Statement

The study protocol was approved by the Tokyo Medical University review board (IRB no.: T2023‐0056). This study was conducted in accordance with the ethical standards of the 1964 Declaration of Helsinki and its amendments.

## Consent

Informed consent was obtained from all participants using the opt‐out method via the institutional website and in‐hospital postings (as it was a retrospective study using information contained in medical charts and computerized records).

## Supporting information



deo270347‐sup‐0001‐Video.mp4

## Data Availability

The datasets generated and analyzed during the current study are available from the corresponding author on reasonable request.
